# Enhancing reproducibility in scientific computing: Metrics and registry for Singularity containers

**DOI:** 10.1371/journal.pone.0188511

**Published:** 2017-11-29

**Authors:** Vanessa V. Sochat, Cameron J. Prybol, Gregory M. Kurtzer

**Affiliations:** 1 Stanford Research Computing Center and School of Medicine, Stanford University, Stanford, CA, United States of America; 2 Stanford University Department of Genetics, Stanford University, Stanford, CA, United States of America; 3 High Performance Computing Services, Lawrence Berkeley National Lab, Berkeley, CA, United States of America; CNRS UMR7622 & University Paris 6 Pierre-et-Marie-Curie, FRANCE

## Abstract

Here we present Singularity Hub, a framework to build and deploy Singularity containers for mobility of compute, and the singularity-python software with novel metrics for assessing reproducibility of such containers. Singularity containers make it possible for scientists and developers to package reproducible software, and Singularity Hub adds automation to this workflow by building, capturing metadata for, visualizing, and serving containers programmatically. Our novel metrics, based on custom filters of content hashes of container contents, allow for comparison of an entire container, including operating system, custom software, and metadata. First we will review Singularity Hub’s primary use cases and how the infrastructure has been designed to support modern, common workflows. Next, we conduct three analyses to demonstrate build consistency, reproducibility metric and performance and interpretability, and potential for discovery. This is the first effort to demonstrate a rigorous assessment of measurable similarity between containers and operating systems. We provide these capabilities within Singularity Hub, as well as the source software singularity-python that provides the underlying functionality. Singularity Hub is available at https://singularity-hub.org, and we are excited to provide it as an openly available platform for building, and deploying scientific containers.

## 1 Introduction

The modern scientist is challenged with the responsibilities of having expertise in a field, procuring funding, teaching, and publishing to maintain a career. The publication that these scientists produce are implicitly expected to be “reproducible”, meaning that they document and make available the methods, data, and tools necessary to repeat experiments and reliably produce similar or identical results. The “reproducibility crisis” [1–3] revealed that a large proportion of publications were not reproducible by other trained scientists. What followed was powerful, proactive action: an investigation and discussion about standards for publication, data sharing, and dissemination of code and tools for reproducible science [1–6].

The scientific community continues to teach and advocate for common practices from software development [7–10] such as continuous testing and version control of code, and storage and sharing of standardized data structures [4, 5, 9, 11–13] to improve reproducibility. Additional assistance is often provided by universities and research institutes by teams of staff dedicated to supporting research computing, but this support is clearly not enough. Arguably, if scientists were afforded tools that enforced reproducible practices that were both easy and enjoyable to use, the difficulties of reproducible research could be ameliorated.

During this same period of time that the scientific community was reflecting on the reproducibility of its work, the enterprise software community was making rapid progress in developing linux containers [4, 14]. Linux containers enable developers to package software dependencies in portable environments that can be seamlessly deployed on different systems. This technology was needed by the HPC (high performance computing) communities, and an inability to provide one of these technologies, Docker [14], in a secure environment led to the development of Singularity containers [15] for scientific compute. HPC communities have, and continue to, bring Singularity to their clusters, meaning that a scientist can package his or her work in a container and run it on any system with Singularity installed, no matter whether that system is a personal computer, a local research cluster, or a cloud environment. The typical workflow entails the user developing the container locally or in an environment where he or she has sudo privileges (see Figure 1 of [15]) and then moving the ready-to-use container to the intended location of use. Here we see a potential bottleneck in the workflow: these containers are too big to host using version control (e.g., Github), and if users can not build a container on the same system where they intend to deploy it, then manually transfer is required, and this becomes quickly burdensome.

Singularity Hub is a modern solution for scientists and developers to quickly build and deploy containers. By way of using version control and webhooks [5, 16], a user is afforded automated container builds simply by pushing a set of instructions to a version controlled repository. Singularity Hub captures metadata about the container and its build, and serves this information, along with the containers themselves, from an interactive web interface and via a RESTful application program interface (API) [17]. The containers are integrated seamlessly into the Singularity [15] command line software, and thus programmatically available for scaled science. This paper will proceed as follows. We will first describe the goals of Singularity Hub, several use cases, and its infrastructure. Next, we will move the discussion to the algorithms provided by the singularity-python [18] software. This robust set of reproducibility metrics has promise to drive reproducible research, and organization of the container ecosystem. For the first time, we study the architecture and metadata about the contents of a container, and use this rich set of features assess the similarity of entire operating systems. Using different levels of reproducibility criteria afforded by the metrics, we propose increased focus on a domain of operating system science.

## 2 Overview of Singularity Hub

### 2.1 Singularity Hub workflow

The Singularity Hub workflow is centered around one function: automatically building containers. It has become common practice for scientists to use version control (Ram, 2013) to streamline collaboration and continuous testing of analysis code. Providers such as Github [19] have recognized this use, and as a result, provide a programmatic method [16] called a webhook to trigger other applications when a change is made to the analysis code. The file that describes a build is traditionally a text file with a set of commands to install dependencies, create content, and set up services. For example, Docker uses a file called a “Dockerfile”, and Singularity has an equivalent file that Singularity Hub expects to be called “Singularity”, optionally with an extension that indicates a tag for the recipe. Thus, the Singularity Hub workflow proceeds as follows:

- User logs into the web portal at https://singularity-hub.org, and authenticates with Github
- User selects one or more code repositories, each with one or more “Singularity” build files, and enables them to build
- Once enabled, pushes to the repository, indicating a change to the code, automatically ping a webhook
- The webhook is received by Singularity Hub to trigger the build of a new image for all updated recipe files
- Each build finishes and images are available programmatically via API and Singularity software

**Fig 1:**
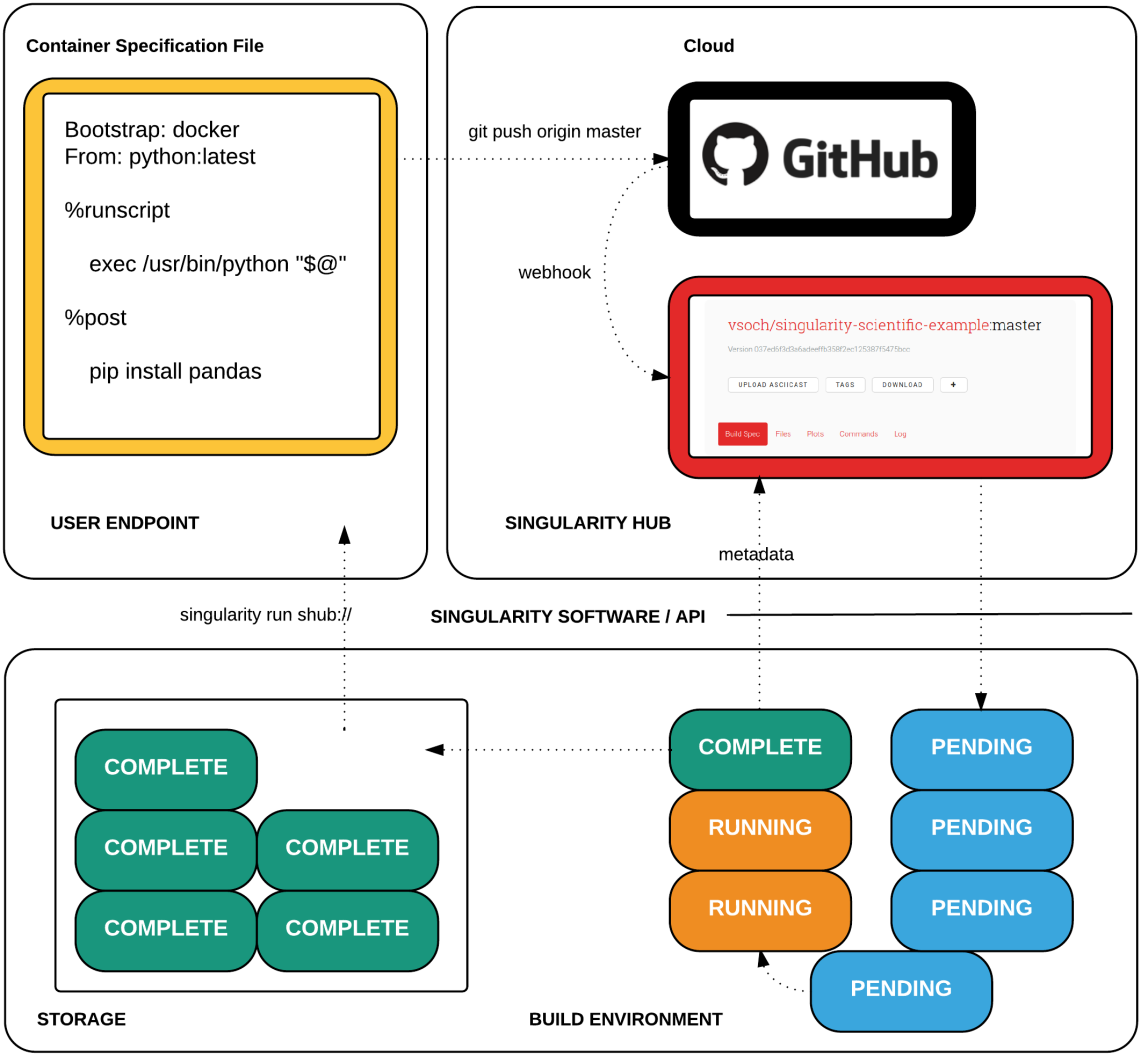
Singularity Hub user workflow. The user creates a specification file to build an image (top left), and pushes this to a version controlled repository (top right). The repository is connected to Singularity Hub by way of a webhook, which sets up and sends a job to a queue to run in the cloud (bottom right). After a series of checks for job priority, the job is deployed to create an instance “builder” that clones the repository, builds the image from the specification file, and sends the completed image to cloud storage (bottom left). Complete metadata is sent back to Singularity Hub to alert the user of the build completion, and the image is available for use by way of the Singularity software and Singularity Hub API.

This process is shown in Fig 1. For the remainder of this overview, we will provide examples using a container in a Github repository under username *vsoch* called *hello-world*. The Github user *vsoch* would connect the repository *hello-world* to Singularity Hub, and in doing so would create a namespace corresponding to a group of images (a collection) associated with the repository. This collection would be called *vsoch/hello-world* to indicate the user and repository from which it is based. The user then develops her code. Each update, done by way of a push [20] to generate a new version (what Github calls a commit [21], would send a message to Singularity Hub to build a new container corresponding to the updated code. If the user *vsoch* wanted to provide multiple different builds in the same repository, she would do that by way of different extensions to the Singularity files in the repository. The user is allowed to have one private repository, meaning a collection of builds that is only accessible to him or her. The choice to allow a user to not share his or her container is done intentionally to be in line with one of Singularity Hub’s goals to support reproducible science, discussed next.

### 2.2 Singularity Hub goals

The application and supporting infrastructure were developed with the goals of making containers moveable, transparent, their builds customizable, and their deployment scalable. Tools are provided that make it easy to compare and assess the reproducibility of containers. All visualizations and functions to build and compare containers provided by Singularity Hub are provided to the user. This open standard and these functions support the larger underlying goal of encouraging data sharing and reproducible science.

#### 2.2.1 Environment agnostic

A core goal of the Singularity software is portability [15], and this extends to Singularity Hub as a registry that serves them. A user should have access to a container served by Singularity Hub from any environment, including a personal computer, local compute cluster, or cloud resource. This is done by way of a RESTful API that serves complete metadata about each container to the Singularity software, which provides a unique resource identifier (uri), *shub://* to retrieve the container itself. For example, a user can interact with the container *vsoch/hello-world* in any of the following ways:

1. singularity pull shub://vsoch/hello-world
2. singularity run shub://vsoch/hello-world:devel
3. singularity run shub://vsoch/hello-world:latest
4. singularity shell shub://vsoch/hello-world
5. singularity exec shub://vsoch/hello-world echo “Hello World!”

In **1**, the user pulls the latest image built from the master branch of *vsoch/hello-world*. The image file is downloaded to the present working directory or a user-specified cache. In **2**, the user chooses to run a particular version of the container, the image associated with the tag *devel* that corresponds to a Singularity recipe with extension devel (Singularity.devel). The “run” command will, by default, run an executable that the user has defined to be an entry point to the container [15]. In **3**, the user chooses to run the latest version of a container, and in **4** the user chooses instead to shell into the container to work on the command line. Finally in **5** the user sends (executes) a command for the container to run. For commands **2**-**5**, the container is downloaded to the image cache defined by the user or system administrator for the Singularity software.

#### 2.2.2 Transparent

For many years, our primary way of interacting with containers and operating systems on remote servers that do not support display tunneling has been by way of a command line. If the container is running, the user can “see” what it contains by shelling into it, and then “mapping” out its contents by using the commands “ls”, and “cd” to list files and change directories, respectively. While providing a build file gives the user somewhat of a look inside of a container by describing the changes made to the base operating system, it isn’t comparable to inspecting a system via software such as a File Manager [22–24]. Singularity Hub aims to be transparent in that it gives the user an interactive visualization to see the inside of every container, and to compare containers.

**Fig 2:**
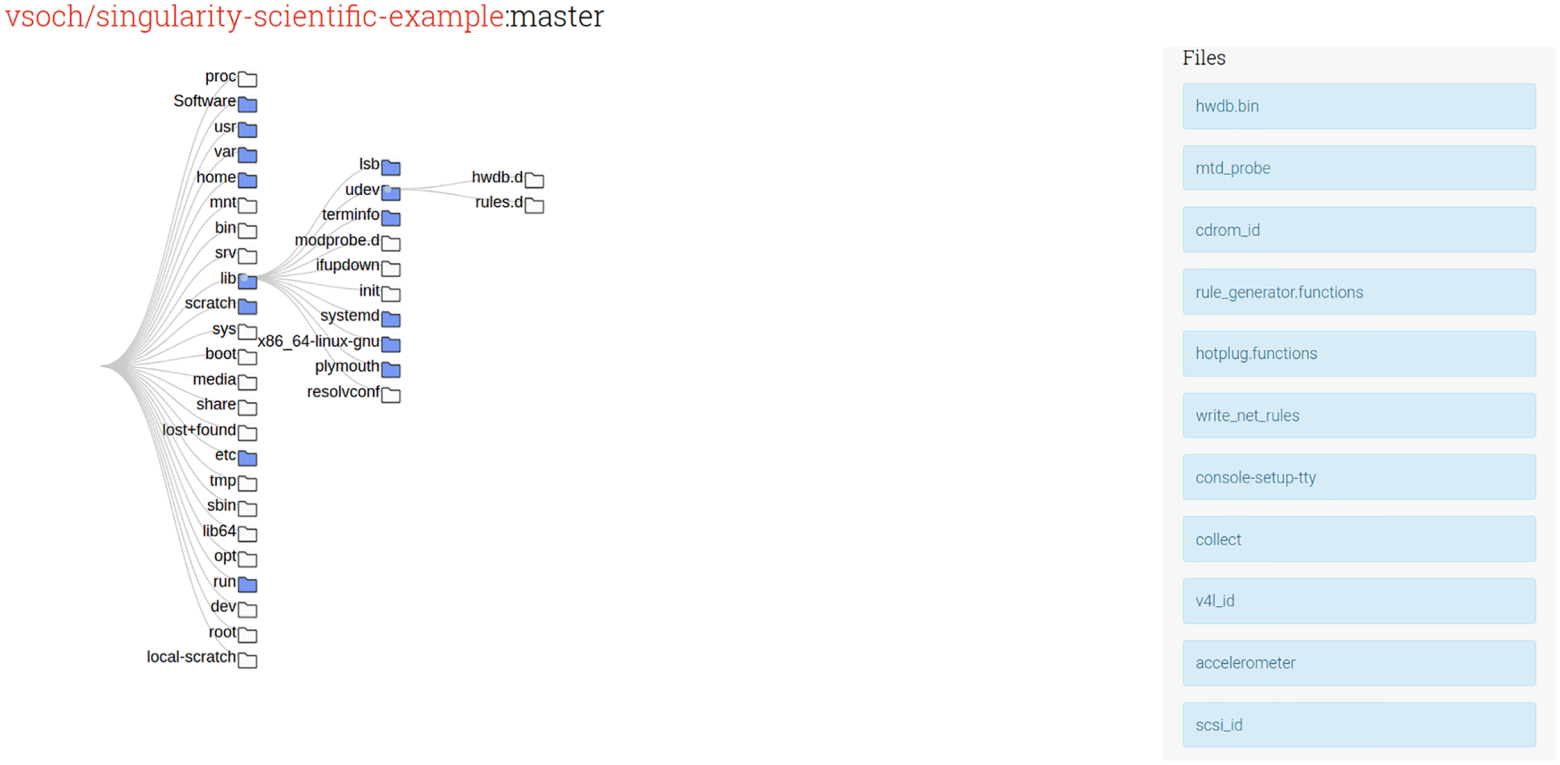
Container tree: Each container is stored with a list of folders and files that render into an interactive tree for the user, both in the Singularity Hub web interface, and on the user’s local command line using the Singularity Hub software.

Singularity Hub shows a container tree (Fig 2) for each image build by generating a hierarchy of folders and files inside for the user to interactively explore using the JavaScript library Data-Driven Documents (D3) [25]. The same functions to generate the tree, and a command line utility to do it locally for an image, are provided to the user by way of installing the singularity-python software [18]. These same lists of files provide a simple means to calculate a similarity metric between containers. By default, the software provides a Jaccard coefficient [26] that is commonly used in information retrieval. We define the similarity score S between sets of files for two containers c1 and c2 as:

$$\begin{array}{c} \frac{2|c_{1}\cap c_{2}|}{|c_{1}\left|+\right|c_{2}|} \end{array}$$ (1)

broadly defined as twice the shared information in the numerator (the intersection of sets c1 and c2), as a proportion of the total summed files (the sum of total files in c1 and c2 in the denominator). While we chose this metric for its simplicity, we provide the raw data in the form of lists of files and folders to the user, also available programmatically via the Singularity Hub API, in the case that the user wishes to derive a custom metric. The user also can take advantage of the functions provided in the same software to derive the lists for images not served by Singularity Hub.

**Fig 3:**
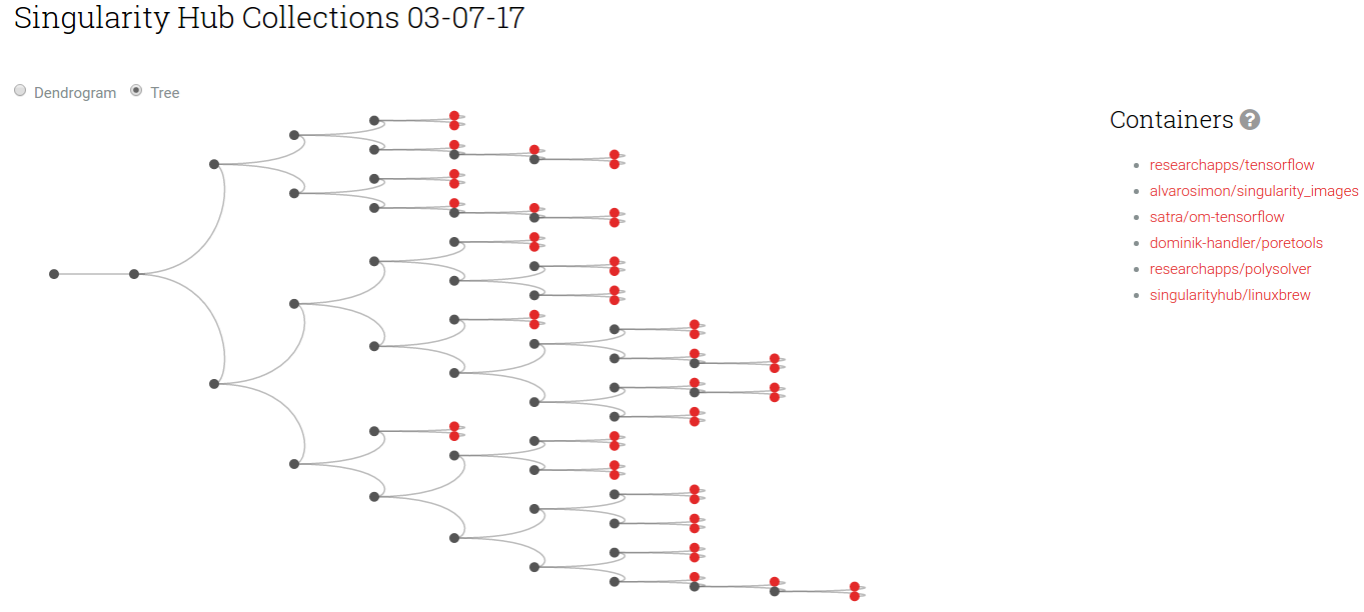
The collection tree provides the researcher with an immediate comparison of the latest version of all containers across Singularity Hub, an easy way to find similar containers using the software and files inside as the metric for comparison. In the example above, a gray node represents a group of containers, and a red node a single container. The user can hover over a node to see all the containers that are represented.

**Fig 4:**
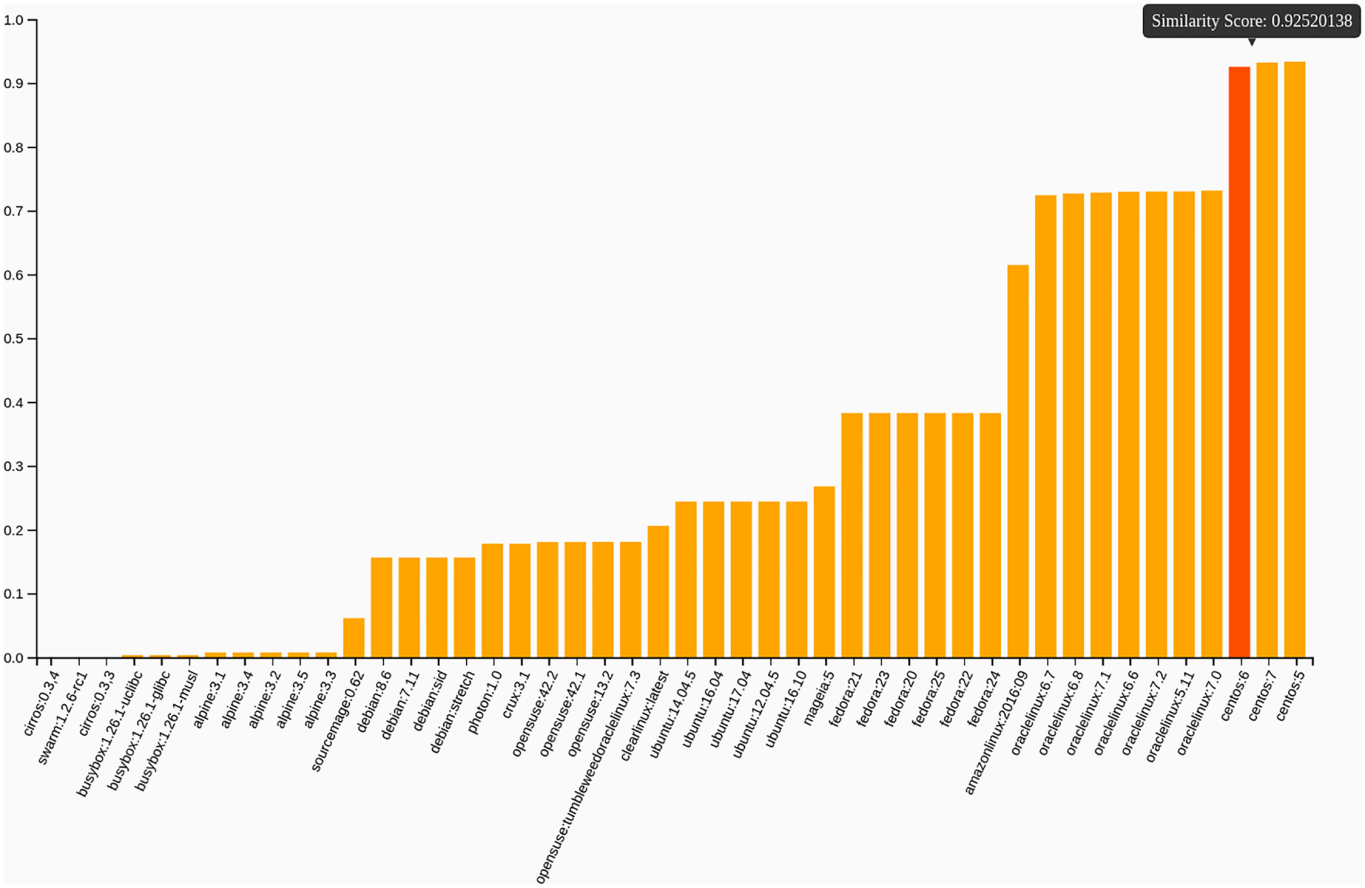
Operating system estimation: Each container is compared to a set of 46 operating systems, including multiple versions of Ubuntu, Centos, Debian, Opensuse, Alpine, Busybox, Fedora, and others. In the example above, the user is highlighting one of the columns to inspect the score, and the build was for a container bootstrapping a Centos 6 image.

This calculation is extended to provide an immediate comparison of the latest containers in Singularity Hub, a “Collection Tree” (Fig 3), allowing the researcher to find containers with similar software. The data structure to generate the visualization is re-generated on a daily basis, and all data archived to provide a rich dataset of a changing container ecosystem for future analysis. Finally, the software provides simple functions to show similar and different files between any two containers, and plots that show the similarity of each container to a set of common base operating systems (Fig 4). A suggested use case would be to find similar containers across Singularity Hub using the Collection Tree view, and then drill deeper with these functions. Importantly, all of these functions and visualizations are available for the user to run and generate locally for his or her research or personal interest by way of the singularity-python [18] software.

#### 2.2.3 Customizable

Early in the development of Singularity Hub, we identified a need that a user may want to customize the builder. For this reason, the builder images are variable within the application. The default behavior of the build is to deploy an instance of size 50GB, however the user can ask the builder to deploy a larger sized instance if more hard disk is needed. We intend to extend this level of customization based on feedback from our users, and allow for custom storage if there is interest. While the current application is deployed on Google Cloud, the code base is modular to make it ready for extension to other cloud environments.

#### 2.2.4 Scalable

The Singularity software uses caching and other strategies [15] to allow for the scaling of image deployment. For a typical use case, the user would make a call to the Singularity Hub endpoint (e.g., *shub://*) and the image would be cached and reused. In the case that one or more images are needed for download concurrently, the storage on Google Cloud was chosen to optimize this functionality. The storage is separate from the web application and image downloads do not drain the resources of the web application server.

### 2.3 Cloud infrastructure

The web application, database to serve it, and storage for images are protected under Google Cloud Default application credentials [27], which are available to the server but not exposed in the web application itself. The database is replicated with a setup called hot standby mode [28] that ensures that all data is mirrored, and an identical instance database available given any failure to the main application. The database, the replication, along with the images and associated metadata are all stored separately and with redundancy, so any failure in one component of the application does not mean loss of all components. The use of Github for version control of build files ensures that, given that some catastrophic event did occur, they could easily be rebuilt by way of rebuilding the files.

Singularity Hub is a Dockerized [14] application, running a secure nginx [29] web server to serve a Python-based (Django) [30] application. The RESTful API follows the Swagger standard [31]. The user is provided with a user guide, a search interface for collections, and a table that lists container collections that is sortable by downloads and user “favorite” stars. Within each container collection is a table of builds, each of which has a tab with example usage (Commands), a comprehensive, colored coded and filterable build log (Log), an interactive bar chart that shows the calculated similarity scores of each build against common operating systems (Plots), a list of labels and internal tags (e.g., file extension counts) derived for the container, and user generated interactive tutorials (Asciinema, YouTube, or external documentation). Across the site, each container build has a small plus (+) button that affords the build to be added to the user’s “Saved Containers”, a drawer on the bottom of the screen where a user can save containers, akin to a cart. Once in the drawer, the user can select containers to view and compare interactively to other containers. This function takes advantage of local storage in the user’s browser, and we hope to extend it to allow the user to perform more operations on single and sets of containers.

### 2.4 Job queue

It was of concern that, given that any authenticated user can build images by way of Github pushes, this ability would be misused in a malicious manner. For this reason, a custom job queue has been implemented that implements the following restrictions and settings:

- Each collection is allowed one running build at a time, and the rest are queued
- A maximum of 15 active and pending builds is allowed, and beyond this number additional requests are ignored until the current queue is processed.
- Any collection that exceeds a rate of 100 builds per hour is disabled
- A total maximum of 5000 jobs is allowed, less than 1/5 of the number that the infrastructure can support
- A global variable can be triggered to turn off the entire process

We tested this queue during our reproducibility demonstration (Reproducibility Demonstration) and verified that each collection only built one container at a time, pushing builds to exceed more than 15 in pending disabled the collection, and triggering the builder to exceed a rate of 100 per hour also disabled the collection.

### 2.5 Builders

The Singularity Hub builders are cloud images that are deployed into instances on demand, and the build is done in an isolated environment from the host. Each is essentially an operating system image, and the most up to date are currently configured with Ubuntu 16.04 LTS and Singularity version 2.4. Each builder is stored with metadata associated to a build family, which generally specifies the builder name, software version, and image size (e.g., singularity-builder-2-4-50gb). In this manner, images of size 50gb and 100gb are provided for each official release (e.g., version 2.4) of the Singularity software. Unless there is a security risk discovered in an older version, these images will be maintained and available for the user to choose from to allow for backwards compatibility as the software develops.

The scripts deployed to the builders to perform the build are sent to the builders by way of the Google metadata API [32], and the programmatic specification of a script allows users to customize the build process to their needs. Other cloud services (e.g., Amazon Web Services) have similar APIs that could also be used in this manner.

### 2.6 Container metadata

The builders collect various metadata about each container (Table 1) including container size, build time, estimated operating system, software and services, container labels and environment, file extension counts, the build specification file “Singularity”, and a complete build log. The metadata is sent to object storage with the containers, and back to the Singularity Hub database, where the user can explore it interactively. Some metrics (e.g., container size and build time) are useful for administrators and users to understand how to optimally store containers, and expected times for building. Other metrics (e.g., file extensions and estimated operating system) have less obvious immediate value, have potential to be a rich set of data for better understanding of scientific software (see Discussion). We provide details about different metrics below, and in Table 1.

**Table 1:** Builder metadata.

Metadata	Description
cloud environment	the cloud environment where the build was completed
builder image	the disk image used as a base for the builder instance
container size	the container size, estimated by the builder, in MB
build time	the build time (in seconds) from start of build to finishing the container.
operating system estimation	the estimated container operating system
file extension counts	a count of files based on extension
apps	a list of modular software defined in the container

In addition to storing parameters about the build itself, the builders collect metrics related to the build process, environment, and the container. The goal is to capture a reasonable amount of information to reproduce and document the build in publications, and any other future use cases.

#### 2.6.1 Container size

The container size is calculated at the end of the container build, and added as metadata to the container itself. It is extracted along with other environment and labels with the “inspect” command applied to the image before sending to storage.

#### 2.6.2 Estimated operating system and software

The Singularity Builder uses the singularity-python software to calculate an estimated base operating system. Once this is done, the files in the base can be subtracted from the list to leave a meaningful set of additions that have been made from the base of the container. This set, akin to a “diff” between two files [33], is used to count file extensions from files relevant to the additions to the container. If the user has installed internally modular applications called Standard Container Integration Format (SCI-F) apps [34] metadata about the apps is extracted as well.

#### 2.6.3 Container log

An essential component of developing a scientific workflow is debugging. Given that Singularity Hub removes the burden of needing to build the container locally, it also takes on the burden of sending back the alarm given that something goes wrong. To meet this need, the Singularity Hub builder uses standard Python logging [35] to print metadata and all command line output to a log file on the builder. The process to complete the build and send the log back to Singularity Hub is executed as a different process from the building command to ensure that regardless of what happens, a signal gets sent back to Singularity Hub to update the user with the status of the build, whether that be a notification that the job completed successfully or that it did not. To make it easy for the user to filter down to key error lines before trying the build again, Singularity Hub color codes the log lines based on the kind of message (debug, error, warning, info, and download) such that the user can click corresponding filter buttons to focus on content of interest. The user can also download the log for further inspection.

## 3 Reproducible products

The infrastructure and underlying data structures and technologies have been designed in a way to incentivize reproducible scientific practice.

### 3.1 Incentives

One of the biggest challenges in the quest for reproducibility is encouraging researchers to share data and code [36–38]. The application supports private collections, meaning that only the owner has access to it, in the case that a container is under development and needs to be kept private before it is published. A user can also “freeze” a container, which is a guarantee to other users that it will not be deleted. However, to reinforce general reproducibility of analysis containers, the user is only allowed one private collection. All users are allowed to create as many collections as desired, coinciding with one Github repository each, and we will only change this standard if it becomes abused.

### 3.2 Version control

Singularity Hub provides version control for all containers by way of Github. Each build is linked directly to a particular commit, build specification file, and branch of a Github repository, and the requirement of a build specification file (the file “Singularity”) is another choice to ensure that the creation of containers carries a level of transparency. Github provides another layer of security in that an exact container version can always be reproduced from its desired build file, given that all dependencies that are downloaded (and not provided in the repository) remain available.

### 3.3 Levels of reproducibility for containers

The preservation of metadata, and coupling of version controlled build specification files with container images is only a starting point for reproducibility. A challenge that we face is the reality that reproducibility is a dimensional definition, and a researcher can choose to operate on several levels. For example, our comparison metric detailed previously (Container Trees and Comparisons) serves as a high level metric to compare containers based on having shared file paths. What this metric does not do, however, is account for the content of the files, which would be a feature desired for an improved assessment of reproducibility. This use case is well suited for a content hash [39–41] that can generate a unique identifier for each file within a container. The strictest level of reproducibility would be an identical container, meaning identical files across it. In this situation, a researcher might build an image, have it copied to another computer, and then verify that all files are represented between containers, and the content hashes are also equivalent. However, if we were to build the same image twice, but at two different time-points, this strategy would not work, because the files would carry different timestamps. This second situation might be called a “replication.” A third situation might be an assessment of reproducibility based on a specific subset of software, or perhaps just the environment or metadata about the container. This use case would desire an equivalent generation of content hashes, but only including a subset of filtered files. Toward this goal, we developed core levels of reproducibility (Table 2), and we apply these levels to the same information coefficient detailed previously (Container Trees and Comparisons) to derive a more robust, level-specific similarity score. We call these filter criteria F, and apply the equivalent similarity metric, but over filtered sets of container content based on the filter to calculate a final score, S:

$$\begin{array}{c} \frac{2|F\left(c_{1}\right)\cap F\left(c_{2}\right)|}{|F\left(c_{1}\right)|+|F\left(c_{2}\right)|} \end{array}$$ (2)

**Table 2:** Levels of reproducibility.

IDENTICAL	This level should be achieved if you download the same image multiple times. All files in the image are used in the comparison.
REPLICATE	The image is generated from the same build file, but at different time-points. File hashes that are different are assessed on the level of the bytes content to address likely different timestamps, and files in temporary (/tmp) and variable (/var) directories are not included.
BASE	The intention of base is to assess if two images are likely to have the same base operating system. Singularity metadata files are ignored, along with files in temporary (/tmp) are variable (/var) directories.
RUNSCRIPT	This level assesses only the content of executable runscript.
LABELS	This level assesses only the content of a labels metadata file.
ENVIRONMENT	This level assesses the content of all files relevant to producing the image environment.
RECIPE	Recipe assesses the content of all metadata about the container, including environment, runscript, and labels (after version 2.3) in the content hash. The base of the container outside this core content is not included.

The Singularity Hub core software, singularity-python, provides and calculates “reproducibility levels” (file and content hashes) for each container build. When comparing two containers, a first filter compares hashes of the files, and a second level filter further assesses results that appear different using content hashes and sizes. Each level maps cleanly to different groupings of files for different build and use cases. The level LABELS is only relevant for Singularity version 2.3.

The filter levels are described in Table 1, and the algorithm to apply them described in the next section.

### 3.4 Assessing levels of reproducibility

Each level of reproducibility is akin to a filter of files within a container to assess if the file should be included, and to what extent. First, we make the distinction between a file hash (a hash of an entire file within the container, including its metadata) and a content hash (a hash of the extracted bytes content from a file without the metadata). As noted previously, the content hash would be needed to assess the example of two files having equivalent content but different timestamps. Next, we define different metrics within a filter, each of which can be thought of as a function that returns a boolean to include the file or not:

- skip files: A list of files to not include, regardless of the regular expression
- include files: A list of files to include
- assess content: A list of files to always compare based on a content hash
- regular expression: A regular expression to determine if a file should be included

**Fig 5:**
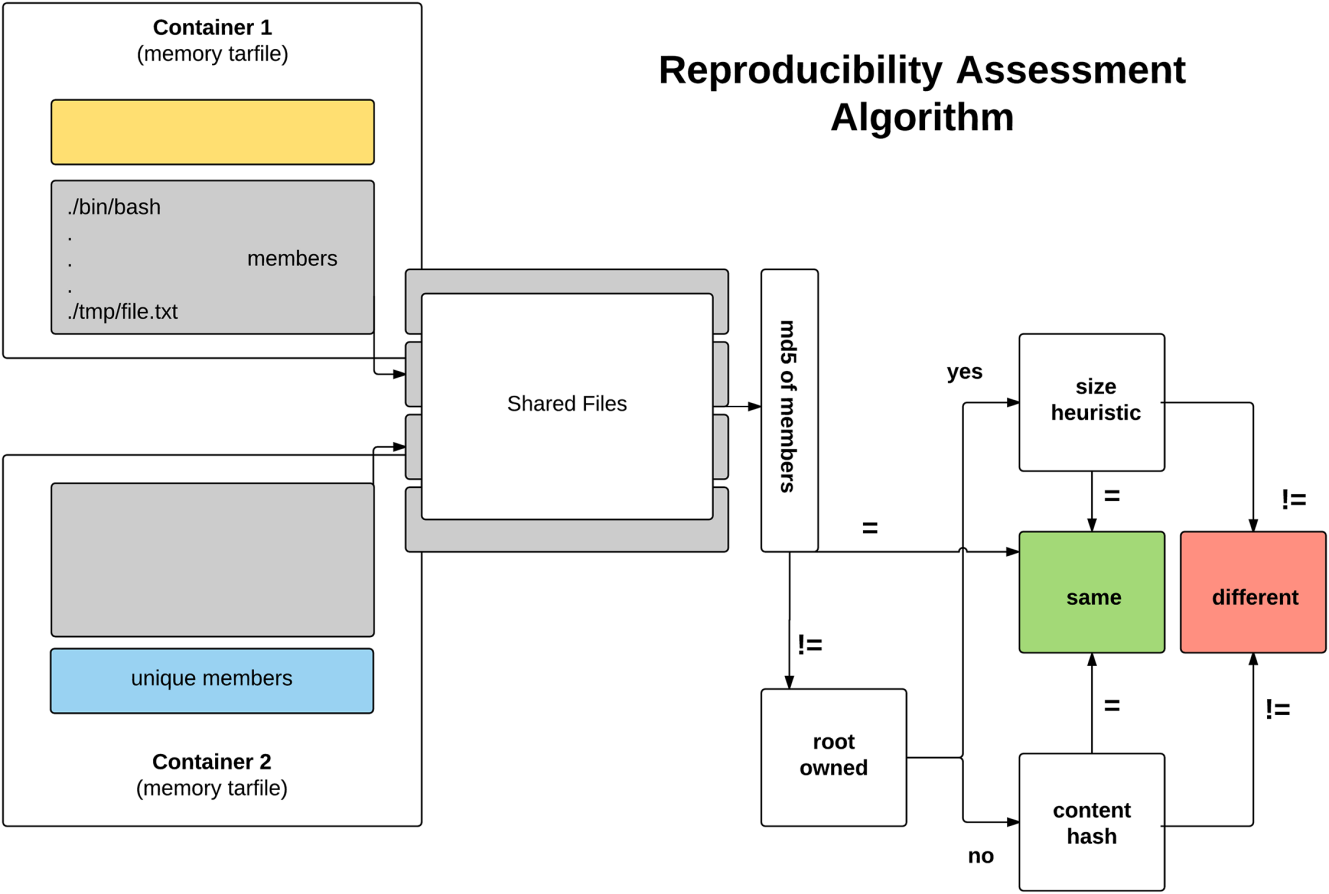
Reproducibility assessment algorithm: A comparison between two containers comes down to comparing the members of the tar stream first based on an md5sum of the file member itself, and then in the case of a mismatch, looking at the content hash (non-root owned) or using a size heuristic (root owned). The final counts of files of overlapping versus different files are then used to calculate an information coefficient using a subset of files particular to a filter (Levels of Reproducibility of Containers) to describe similarity of the two containers.

The filtering algorithm, for any specified level, considers each of these specifications, and toward the goal of making it usable for the user without sudo privileges, also takes the file permissions into account when further assessment is needed. The algorithm proceeds as follows, and is illustrated in Fig 5). Each container has its contents streamed into an in-memory tar. For each file in the tar objects, called “members”, the md5 hash is calculated using hashlib [39]. These hashes are for the file objects, including the timestamps and metadata for their respective file. The final lists of files, along with their hashes, are then compared between the two containers. Files that are unique to each container (present in one but not the other, represented in yellow and blue sections of Fig 5) are considered to be in the difference of the two sets. Within the set intersection (Shared Files), the md5 sums of the files are compared. Files that match are called “same” (green). Files that are not the same on the level of the file hash are taken to the next level of assessment, which first looks at root ownership. If the file is not root owned, then cat [42] is used to extract the bytes content, and we generate two hashes for final comparison. In the case of root ownership, we cannot use cat to extract the contents, and make the final comparison based on the size of the file (size heuristic). Equivalent sizes are considered equal, and the file is added to same, and different sizes classify the file as belonging to different. In the case of a non-root owned file, we generate a hash from the bytes, and then again compare the hashes, for placement into respective groups (Fig 5).

In context of the equation described previously, the groups “same”, “unique” and “different” map cleanly to our equation:

$$\begin{array}{c} S=\frac{2|(same)}{|c_{1}\left(unique\right)|+|c_{2}\left(unique\right)+different|} \end{array}$$ (3)

These criteria are programmatically accessible by way of data structures included with the singularity-python package [18], and customizable by the user. The software can either generate hashes for all files pertaining to a level (in the example above to compare two containers) or add all files to one summarized hash to describe a single container.

Functions are provided to assess two containers, or to derive data structures with hashes, sizes, and information about root ownership. This final function is intended for those users who are interested in using the software for their own research about reproducible software. We provide examples and the complete software [18] for this use case.

## 4 Reproducibility demonstration

### 4.1 Build consistency

To demonstrate the ability of Singularity Hub to build images at scale, we selected a subset of scientific analyses that had Singularity containers built for previous work (Table 3) and used the web interface to build them automatically on Singularity Hub. The containers include a standard “Hello World” example (*vsochsingularity-hello-world*), a more robust machine learning prediction tool for physics (*researchapps/quantum_state_diffusion*) and a published clinical algorithm to predict pulmonary embolism from radiology reports (*vsoch/pe-predictive*) [43, 44]. For each container, we wrote a simple python script (included with the code in the respective repositories), to make a trivial change to an echo statement in the build file and automatically push to Github to trigger a new build. We did this 100 times per container, with a delay (10, 25, and 30 minutes for each analysis container group, respectively) between pushes to ensure no more than 15 pending containers at once. For each of these builds, our goal is to assess consistency of the build itself. If containers are built consistently, we would expect to see no significant differences in the sizes of our containers, which are estimated by the builders by first building the image into a folder. We would also expect to see small variation in build time (the time from the start to finish of the build, not including time to initialize and shutdown the builder) likely due to instance specific variables like network connectivity and latency.

**Table 3:** Analysis repository collections.

Singularity Hub Collection	Github Repository
vsoch/singularity-hello-world	https://www.github.com/vsoch/singularity-hello-world
vsoch/quantum_state_diffusion	https://www.github.com/vsoch/quantum_state_diffusion
vsoch/pe-predictive	https://www.github.com/vsoch/pe-predictive

Analysis repository collections built on Singularity Hub for demonstration of reproducible builds. For each Github repository specified, a collection was generated on Singularity Hub, and equivalent images built 100 times to demonstrate the reproducible build process.

For assessment of build consistency, ipython notebooks and scripts used for the assessment are provided as examples with the Singularity Python software [45].

### 4.2 Reproducibility metrics

To assess our reproducibility metrics, we would want to demonstrate that the levels can accurately measure known criteria about different sets of containers for which we know some level of ground truth (Ground Truth Assessment), that container scores produced by the levels represent a humanly interpretable range between not similar at all (0.0) and completely identical (1.0) (Metric Interpretability), and that the metrics can be used for new discoveries and comparisons between operating systems that were not previously possible (Potential Assessment).

#### 4.2.1 Ground truth assessment

The reproducibility metrics, if they meet their intended purpose of providing a computational way to assess similarity of containers, should operate as expected given a set of containers that meet a certain criteria. Toward this goal, we define three sets of 100 containers (total = 300), and criteria expectations for each, as follows:

Singularity Hub Replicates: These containers represent an image built from an equivalent build specification file, one hundred times, on different hosts (each being a Singularity Hub cloud builder). Specifically, we used the *vsoch/singularity-hello-world* [46] collection mentioned previously (Build Consistency). We would expect a single container compared against the others in the set to be assessed as equal on the levels of RUNSCRIPT, LABELS, ENVIRONMENT, BASE, and REPLICATE, but different on the level IDENTICAL.

Local Replicates: These images represent an image built equivalently from the same build file, but on the same host. We used an equivalently simply build specification file, and built these images on a local machine with the same operating system as the cloud builders (Ubuntu 16.04). We would equivalently expect any image from this set assessed as equal on the levels of RUNSCRIPT, LABELS, ENVIRONMENT, BASE, and REPLICATE, and different on the level IDENTICAL, however we would expect these images to have a higher similarity score on this last level by way of sharing the same host, which would mean more shared mounts, and cached layers used to generate the images.

Clones: We selected one image from the first *Singularity Hub Replicates* group to download one hundred times to asses our strictest level of comparison, IDENTICAL. The clone images should have the same summary hash on every level of comparison.

#### 4.2.2 Metric interpretability

A successful metric should provide a humanly interpretable score between 0.0 (completely different) and 1.0 (exactly identical) when used to compare two containers. Specifically, the metrics should return a score of 0.0 given that there are no shared files between two containers, a score of 1.0 given that they are identical, and a gradient between those values for intermediate states. To test this, we chose five core open source operating systems (Alpine, Debian, Ubuntu, CentOS, and Busybox), and generated Singularity containers with a complete installation of the operating system for each. The containers were generated by way of creating empty images, and then importing Docker base layers for the latest version of each operating system into each. For example, the command to create the Ubuntu image is the following:

**sudo singularity build ubuntu.img docker://ubuntu**

For each of these containers, we conduct an analysis to assess metric interpretability as follows. We define C1 as the container that has all files present for the operating system. We define C2 as a separate container with the same operating system, but with a portion of files removed. We define L as a list of files in the full container, sorted by the time-stamp from oldest to newest. Given a container has a total of M files, we calculate the comparison between C1 and C2 for a range of N = 0:M across reproducibility levels, removing file in L at index N at each step. This comes down to removing the newest files first to generate a slowly changing gradient of similarity that should, if the metrics and methods are sound, start at 1.0 (a comparison between two identical file systems each with M files) and finish at 0.0 (a comparison between a full operating system with M files and an empty container). We would expect the scores for the reproducibility levels assessing the content of specific files (RUNSCRIPT, and LABELS) to remain constant until the files are removed from the container (which happens almost immediately given sorting based on time-stamp), and we would expect the levels REPLICATE, BASE, and IDENTICAL to slowly decrease in similarity as files are removed.

#### 4.2.3 Potential assessment

Finally, we would want to show that the reproducibility metrics have value to provide new insights about containers, and allow for comparison of unlike operating systems, which was not previously common practice. We were also interested in assessing how well our Singularity Hub comparison heuristic (Container Trees and Comparisons) that used file paths alone reflected the more robust metric (Assessing Levels of Reproducibility) that also accounted for hashes. Toward this goal, we computed pairwise comparisons on the level of REPLICATE for all publicly available scientific container in Singularity Hub. We used representational similarity analysis [47] to compare this matrix of similarity scores to one generated with our similarity heuristic. Similarity of these two matrices would suggest that the comparison of file paths alone can be useful to reflect the more robust reproducibility metrics. This similarity matrix would also allow us to see commonalities between the scientific containers that are not readily apparent.

For all analyses above that required Singularity Hub images, we used the Singularity Hub API via the singularity-python software to download container images and metadata. For analyses that required local builds, we used this same software as a wrapper to the command line Singularity software [15]. The reproducibility assessment functions were provided in the *reproduce* module within singularity-python.

## 5 Results

### 5.1 Build consistency

If containers are built consistently, we would expect to see no significant differences in the sizes of our containers, and this turned out to be the case. For each of our containers builds, all 100 builds generated images of the same size (80, 120, and 75 GB for each, Table 4) and while there was some variance in build time, when assessed by a 1-sample T-test there were no values that different significantly from the mean (p = 1.0, T-statistic = 1 for all builds). A summary of our findings for build metrics is included in Table 4, and an ipython notebook for generating and viewing plots of the result is available [45].

**Table 4:** Builder consistency.

Size Mean (GB)	Size STD (GB)	Build Time Mean (sec)	Build Time	STD (sec)
vsoch/singularity-hello-world	80.0	0.0	12.31	1.14
researchapps/quantum_state_diffusion	120.0	0.0	649.88	41.05
vsoch/pe-predictive	75.0	0.0	2687.07	208.28

Singularity Hub Builder Consistency: When assessing the builders for build time and size of resulting containers, across N = 100 containers for each collection, we see no variance in build size, and no significant difference in build times (p = 1.0).

### 5.2 Reproducibility metrics

#### 5.2.1 Ground truth assessment

Singularity Hub Replicates: Using a set of 100 containers each generated with separate Singularity Hub builders for the *vsoch/singularity-hello-world* collection, we compared a randomly selected container against the rest for the reproducibility levels RUNSCRIPT, ENVIRONMENT, BASE, and REPLICATE (S1 Table). In order to trigger the builders, we had to change a trivial space in the runscript to make a Github commit, and were pleased to see this reflected in the results. For half of the containers with the equivalent runscript, the reproducibility level RUNSCRIPT was 1.0, and for the other half with a different runscript, it was 0.0. This trend was the same for this same set of half of the images for RECIPE, decreasing the score (0.8) to reflect the different runscript, and for REPLICATE (0.99) to reflect the one different file. Given the building on different hosts, the IDENTICAL level was always slightly less than 1.0 (0.99). In this case, the different files between the images were those related to package manager downloads from different mirrors and those related to information about the host (e.g., /var/hosts). The level that assessed the containers as most similar was BASE (1.0 across all containers).

Local Replicates: For our equivalent collection of containers built on the same host, as expected, we saw scores of 1.0 (highest level similarity) on the all levels except for IDENTICAL, as the image files carry different timestamps. However, by way of sharing the host, we found the IDENTICAL score to be slightly higher (0.9956) than the assessment for the Singularity Hub replicates (0.9952), a result that is likely not significantly different, but expected for the levels. Complete results are included (S2 Table).

Clones: Assessment of the clone revealed scores of 1.0 across all reproducibility levels (S3 Table), an expected result as the image file is exactly the same, simply downloaded many times.

#### 5.2.2 Metric interpretability

**Fig 6:**
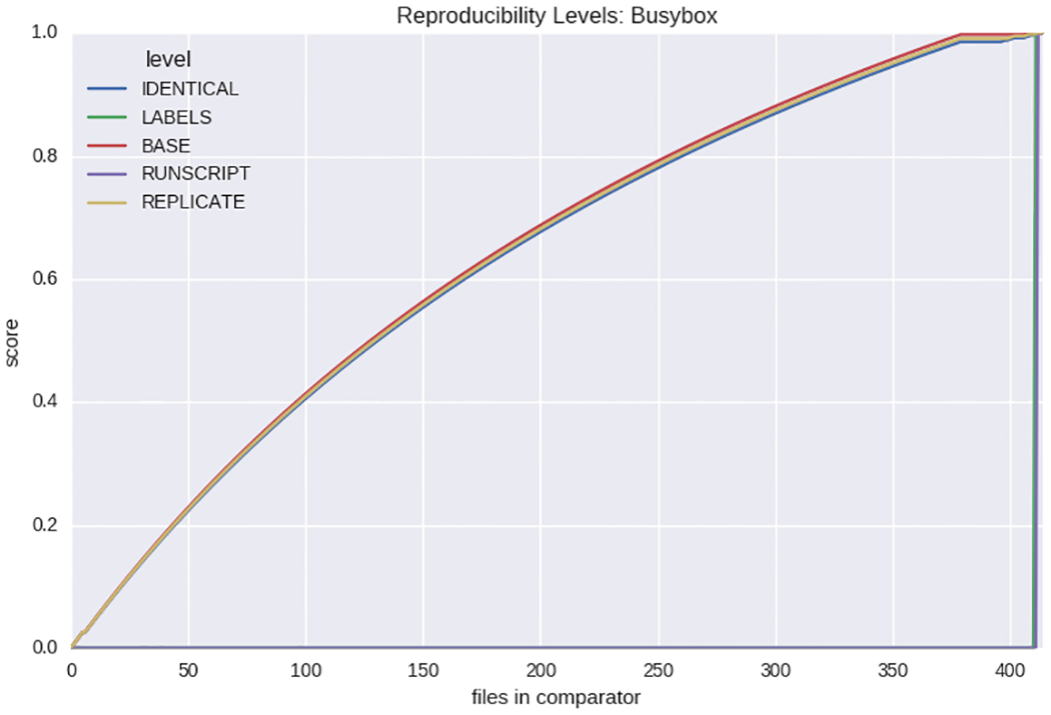
Reproducibility level gradients: As an assessment of metric interpretability, we calculated comparisons for each metric for a complete operating system (e.g., Busybox) against all versions of itself with one file removed until no files remained. As we remove files starting with the newest based on time-stamp (from right to left) similarity decreases between the full container and it’s comparator until we reach a score of 0.0. The metrics for RUNSCRIPT, LABELS, by way of being the newest files in the image, return a perfect score of 1.0 given that they are present in both images, which only occurs at the far right of the plot.

To assess if our metrics could accurately reflect a dimension of intuitive similarity scores, we calculated comparisons across levels to compare base operating systems (Alpine, Debian, Ubuntu, CentOS, and Busybox) against all versions of themselves subsequently removing one newest file until the images were empty. The scores passed our criteria of returning a value of 0.0 when no files are shared, and a value of 1.0 when the images are identical across all metrics. An example evaluation for the Busybox operating system is included in Fig 6, and additional operating systems are provided (S1–S4 Figs).

#### 5.2.3 Potential assessment

**Fig 7:**
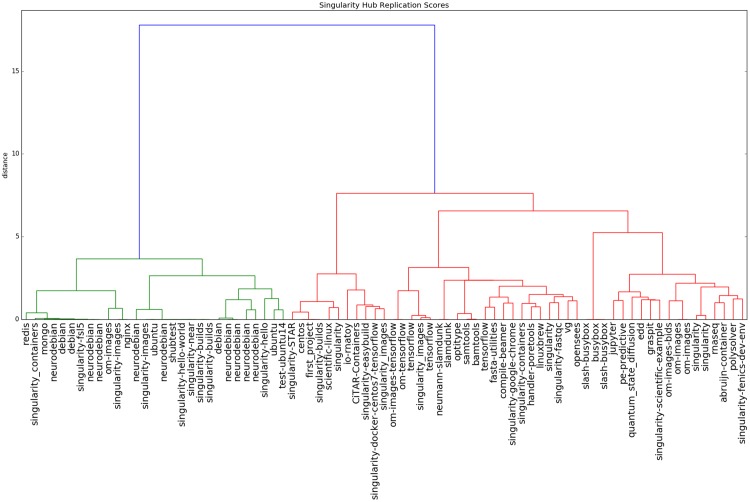
Hierarchical clustering of Singularity Hub public images (accessed March 28, 2017) with Ward’s metric. We observed that containers generally clustered first on the level of operating systems (dark blue) in the order of Debian, Ubuntu, and then CentOS and Busybox.

We calculated pairwise comparisons using the reproducibility level REPLICATE for all public images in Singularity Hub (N = 79), to generate a pairwise similarity matrix (S1 Data and https://plot.ly/vsoch/21). We found that containers clustered first on the level of operating systems, and then by common software (Fig 7). Representational similarity analysis (RSA) of the pairwise comparisons using the reproducibility level REPLICATE against pairwise comparisons done using file paths resulted in a score of of 0.74, suggesting that a comparison of file paths can capture some, but not all information done with a more rigorous comparison.

## 6 Discussion

### 6.1 Reproducible building

We have demonstrated that the Singularity Hub builders are reliable to produce replicates of an image, and an identical image that can be downloaded multiple times. We have also developed and provided metrics for scientists to use to compare entire containers based on a custom level of reproducibility. Each of these levels carries suggestions for intended use.

### 6.2 Best practices for reproducibility levels

IDENTICAL: The strictest level of reproducibility comes down to the scientist providing a download of the exact container that was used for his or her work, which is a strong use case for Singularity Hub. Under these conditions, the container in Singularity Hub can be referenced in publications, linked to in web content, and available immediately on the command line via the Singularity client. Any assessment of replication of this container, given that the download is complete and successful, will return a score of 1.0 using our levels. If the scientist is interested in providing a container at this level, he or she can use the singularity-python functions to provide a hash of the file itself, which would be reliable to assess the file.

REPLICATE: If the scientist is interested in comparing containers that, although they might have been built using the same specification, were built on different hosts, or at different times, we recommend using the reproducibility level REPLICATE. A replicate will allow for different timestamps and files relevant to host information and temporary.

RECIPE: The level RECIPE and the levels that it includes (ENVIRONMENT, LABELS, and RUNSCRIPT) are useful if the scientist is only interested in assessing the intended execution (RUNSCRIPT) or environment (ENVIRONMENT) or metadata (LABELS) about the container. For example, containers with entirely different operating systems may have the same execution statement to run a scientific software. For these levels, akin to IDENTICAL, the user is able and encouraged to calculate summary hashes for later comparison because the data structure has the “assess content” field that tells the software to always use the bytes content for a specific set of files, and thus the hash generated will always reflect the content of the file and not be relevant to the time-stamp.

BASE: Using the level BASE is the best way to assess the similarity of two containers regardless of the generator’s intended use. For example, two researchers might start with equivalent containers, and then have custom environment exports and runscripts to specify the container for a particular use case. While the levels IDENTICAL and REPLICATE would reflect these similar bases, they would do so less accurately than using BASE alone.

CUSTOM: The user is also encouraged to generate a custom level given that the reproducibility levels provided do not meet his or her needs.

With the singularity python-software, we hope that the user is empowered to choose the level of reproducibility that is most appropriate for his or her use case, and we provide these suggested practices to advise about when each level is most useful. For the reason that there are many use cases and no “one size fits all” solution, Singularity Hub purposefully does not bias the user to use any one level.

### 6.3 Reproducibility level dimensions

While an assessment of how the removal of different files, or further, groups of files is outside the scope of this paper, it is an interesting example of the kinds of work that are afforded by availability of these tools. For the first time, we can quantify the differences in a software or file footprint, whether that be a python installation, a python module, or even an extension of file. While we chose to look at files across an operating system, it is clear that this same procedure, when applied to different versions of software, or software compiled in different ways could provide detailed measurements that give insight to software optimization. For example, is equivalent software on different operating systems comparable, and by how much? Are analysis output files generated with equivalent seeds on different operating systems truly identical? Are they replicates instead? We would want to extend this kind of comparison to look at the footprint of software, and data files, and start work that might better map the network of interactions that happen within an operating system.

#### 6.3.1 Potential assessment

We were surprised at the extent of information that we were able to learn about operating systems from the plots of our reproducibility metrics. For example, across operating systems we were able to quickly determine the general proportion of symbolic links by finding constant trends in the plot (for an example, see S2 Fig for the Alpine Operating System). Since symbolic links are not accounted for in favor of the actual files, their removal does not change the score. When looking at the clustering (Fig 7) we were excited to see clear structure in even a small dataset, and hope to see future analyses with more containers across different levels.

### 6.4 Limitations

#### 6.4.1 Reproducibility metrics

The pairwise calculation to compare many containers is computationally not fast (2-5 seconds for a container across all levels, depending on the size). We first developed the software using lists and later increased speed by 10X by using sets. A small container of approximately 400 files can take 1.5 to 2 seconds, and a larger container of over 10K files is closer to 5 seconds. The processing time is due to the need to read bytes content directly for all files that are originally assessed to be different based on file hash. For the user wanting to make a small number of comparisons on the fly or locally, our software is reasonable to use, and for the user wanting to do many comparisons, we recommend the standard approach to scaling by way of running jobs on a cluster or in parallel, or using the singularity-python functions to cache hashes. We thought about using another heuristic that would not require reading bytes to assess the file, such as size, and while we think the comparisons would likely be accurate, they would have the potential to produce false positives (files assessed as equal based on size but are not truly equal in content).

Our current approach does not do well to identify two identical files that are named differently, whether they be located side by side in the same directory or in different locations in the image. For most software installation, we recommend to the user to ensure installation in standard system locations by way of using package managers. However, we think it would be useful to be able to “sniff” a container to assess if it contains a software of a user’s choice (for example, it might be useful to know that Python anaconda version 2.0 is installed in two containers, albeit in different locations). We would consider this kind of comparison, for one file or a set, a more “functional” comparison, as using “python” in the image would have the same desired execution given that the installations are on the path. Further, we would want the user to be able to look for an entire set of files pertaining to a particular software installation, and we would want to be able to map a standard location in one operating system to a (possibly different) standard location in another. It could also be useful to alert a user when a container has more than one version of software. For example, with Python it is common to mistakenly install the same module to multiple locations, or different versions to the same installation, and then produce errors related to finding the wrong version first via the python path. We are thinking more about these problems, and currently working on functions that might help to address them.

In this same line of thought, often what is most interesting about a container isn’t the software installed from package managers, but the custom software created by the scientists. Toward this goal, we hope that Singularity Hub, by way of capturing signatures of common software, can then use these signatures to identify unique software. A more robust metric would, akin to how we do with estimation of operating system, first be able to subtract out a base operating system. A more robust metric might also apply a weighting to our current algorithm to calculate the similarity score, placing higher weight on custom software.

Another limitation is a lack of normalization for software size. Currently, metrics of similarity are based on overall similarity of files, and don’t account for a software packaging having more or fewer files. For example, a series of files that are not relevant for the function of the image, perhaps leftover output files, would be included in the total (in the set represented in one image but not another) and make the images look very different even if the core software (the function of the image) was equivalent. This could be addressed on a user level by creating a custom filter that would skip over the pattern of output files by regular expression, but would need to be thought about in advance. These custom sets of files are an important feature to think about, because it should be the case that groups of meaningful paths (software) can be integrated into the algorithm. To allow for the user to do these custom analyses and exploration of custom metrics, singularity-python allows the user to derive the intermediate hash values and inputs to our algorithm so he or she can develop more robust or better customized metrics.

### 6.5 Future development

Operating system science must focus on the assessment of not only operating systems, but software development practices, organization of systems, and changes over time. An operating system is more than a static structure of files and folders—it is a phylogenic tree where file timestamps, hashes, organization, sizes, and extensions carry rich information about what we, as humans, value in our daily lives. It could be that an older file or set of files reflects having strong structure or practices that have withstood the test of time. It could also be the case that there is a subset of these older files that are carried around but unnecessary, or perhaps executables that have great potential that have been forgotten. Comparison between file systems could show us that base operating systems are in fact very similar or different than we imagined, and might open the door to thinking about how machine learning could be used so that operating systems can define themselves, optimized for some purpose. With enough data, we might even be able to infer different types of human behavior and personality based on preferences for naming, storing, and changing our systems. The potential for this new area of research is tremendous, and we will finish this paper with some final thoughts about these developments.

#### 6.5.1 Understanding scientific software

The landscape of scientific software is poorly understood. It is not clear what makes good scientific software, or even what metrics we might use to quantify “good.” If we are to optimize our development of scientific products, an understanding of what dependencies are commonly used, changes over time, and patterns in both development and the code itself are observed. We might also want to observe the way that operating systems are organized to properly define best practices. By preserving these metadata about scientific containers, and treating file hierarchies and extension counts as features, available with functions to our users, the developers of Singularity Hub hope to encourage work in this emerging domain. As it becomes more common practice to see research software engineers, we hope it to become common practice to quantify development procedure, code quality, and ultimate success of the product, as this is the local means to better understand where there is room for improvement.

#### 6.5.2 Integrations

To introspect on the future of scientific workflows and the Singularity Hub container registry, we hope to see, and have plans to develop, integrations that work with Singularity Hub. By way of making containers accessible via the Singularity command line software, Singularity containers served in the Singularity Hub registry can already be plugged into workflow software [48] and traditional job managers [49, 50]. We have vision for containers to provide science as a service, meaning that robust, standardized, and collaboratively developed analysis containers can be served on Singularity Hub and used by all, including those that may not have the expertise to develop the tools. Given better sharing and dissemination of these containers, we can have more specialization in the scientific process. Those with strong computational backgrounds and interest can optimally develop software, diagnose problems, and innovate methods, and those with invaluable domain knowledge and vision can focus on the asking and answering of scientific questions. While prowess in software development is a benefit for the scientist, it should not be a base requirement, and further, we would argue that each researcher independently developing custom workflows unique to an individual project and publication is inefficient.

#### 6.5.3 Science as a service

These components come together to support the idea of science as a service, or making it easy to plug data into analysis pipelines, run them, and return a result. We can extend this idea to describe how a more fluid and seamless pipeline, made possible by software integration with container registries, can fundamentally change the way we understand the dissemination of scientific information. Instead of the product of a researcher being a scientific result, associated with one point in time and a hard copy of a manuscript, the products of science become the workflows. If each workflow understands the kind of data inputs it can accept, it could be possible to re-run an analysis with new data inputs, produce an updated result, and thus update our understanding of the world. We may, under this kind of reality, expose an elusive concept about our reality that we have historically not been able to see: that although there may be some fundamental truths, many phenomena, tangible things, and ideas that are understood as truths can change.

## 7 Conclusion

We have built, deployed, and will maintain Singularity Hub as a registry for containers for scientific reproducibility. Our reproducibility level tests show that utilizing containers can enable researchers to distribute reproducible computing environments along with source code and primary data to make every aspect of computational analyses reproducible and archivable. To the best of our knowledge, our work is the first of its kind to provide means to do a comprehensive assessment of similarity between containers and operating systems. We are hopeful to incite more interest in this area to better computationally capture and define metrics for reproducible software, and develop tools and best practices for it. We encourage researchers of all computational skill-levels to consider utilizing containers to encapsulate their computing environments, and to discuss container options with IT support staff if none are currently available to them. We direct the user to the README and examples folder in the singularity-python software [18] to learn more, and look forward to sharing our work using this tool.

While we cannot predict if local HPC versus cloud infrastructure will be a preferable approach for researchers, the need for institution-based compute will likely endure in parallel with cloud use, and so an optimal strategy is to develop scientific products that move seamlessly between these places. Singularity containers, provided easily by the Singularity Hub registry, make this possible without needing to worry about software dependencies, or user and environment discrepancies.

## Supporting information

S1 Fig Reproducibility level gradient Debian. We calculated a comparison of the Debian base operating system against all reduced versions of itself, removing files one at a time sorted by earliest first (files removed). Files removed between index 3904 and 4441 were locale relevant files in /usr/share/zoneinfo, and their removal did not change the score because they were symbolic links that are accounted for by way of comparison of the files they link to.
(PNG)

S2 Fig We calculated a comparison of the Alpine base operating system against all reduced versions of itself, removing files one at a time sorted by earliest first (files removed). The Alpine operating system is interesting in that it consists primarily of symbolic links, so that after the Singularity metadata files are removed from the comparison (right side of plot) along with other shared files, the score remains constant as the symbolic links are removed. This is a good example for how the metrics can give insight to operating systems, as we did not know this about Alpine before doing the assessment.
(PNG)

S3 Fig Reproducibility level gradient Ubuntu. We calculated a comparison of the Ubuntu base operating system against all reduced versions of itself.
(PNG)

S4 Fig Reproducibility level gradient CentOS. We calculated a comparison of the CentOS base operating system against all reduced versions of itself.
(PNG)

S1 Table Singularity hub replicates. Comparison results across reproducibility levels for a randomly selected container from the *vsoch/singularity-hello-world* collection against all other containers in the collection.
(TSV)

S2 Table Local replicates. Comparison scores for containers built on the same host with expected scores of 1.0 (highest level similarity) for all levels except for IDENTICAL (the image files carry different timestamps).
(TSV)

S3 Table Singularity hub clones. Assessment of the clone revealed scores of 1.0 across all reproducibility levels, an expected result as the image file is exactly the same.
(TSV)

S1 Data Singularity hub similarity matrix. Pairwise similarity matrix for comparisons using the reproducibility level REPLICATE for all public images in Singularity Hub (N = 79) at the time of the analysis.
(TSV)
