# Supplementary figures and images for: Enhancing reproducibility in scientific computing: Metrics and registry for Singularity containers
Source: PLoS One. 2017 Nov 29;12(11):e0188511. doi: 10.1371/journal.pone.0188511 (PMC5706697; doi:10.1371/journal.pone.0188511)

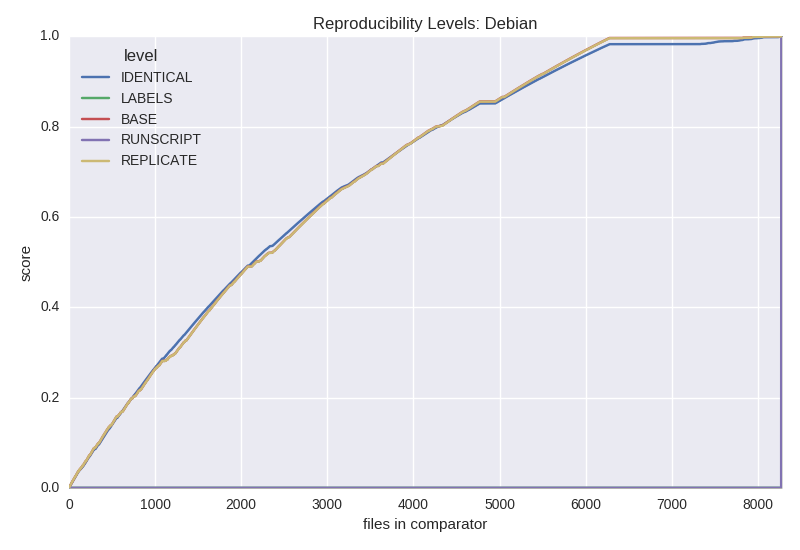

Supplement: S1 Fig — We calculated a comparison of the Debian base operating system against all reduced versions of itself, removing files one at a time sorted by earliest first (files removed). Files removed between index 3904 and 4441 were locale relevant files in /usr/share/zoneinfo, and their removal did not change the score because they were symbolic links that are accounted for by way of comparison of the files they link to. (PNG) [file pone.0188511.s001.png]

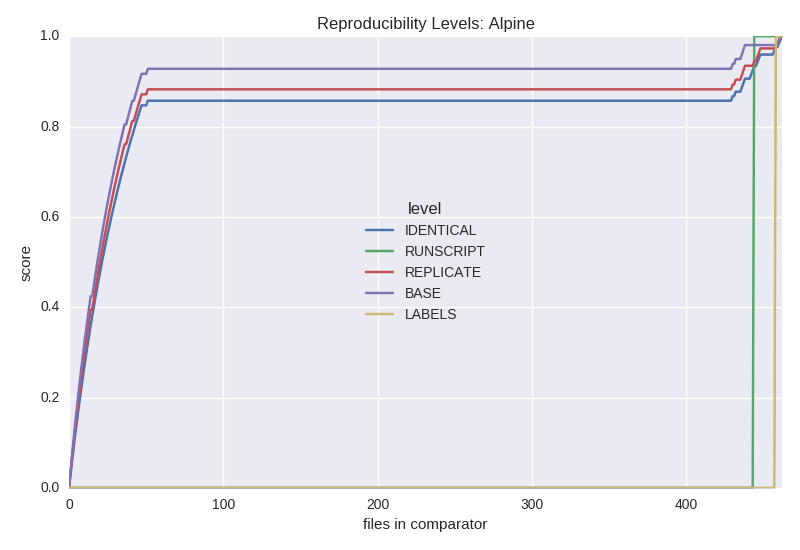

Supplement: S2 Fig — The Alpine operating system is interesting in that it consists primarily of symbolic links, so that after the Singularity metadata files are removed from the comparison (right side of plot) along with other shared files, the score remains constant as the symbolic links are removed. This is a good example for how the metrics can give insight to operating systems, as we did not know this about Alpine before doing the assessment. (PNG) [file pone.0188511.s002.png]

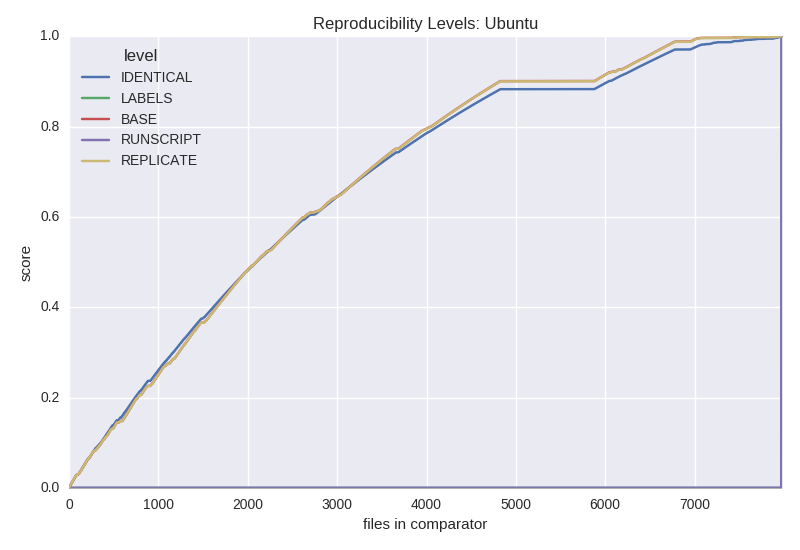

Supplement: S3 Fig — We calculated a comparison of the Ubuntu base operating system against all reduced versions of itself. (PNG) [file pone.0188511.s003.png]

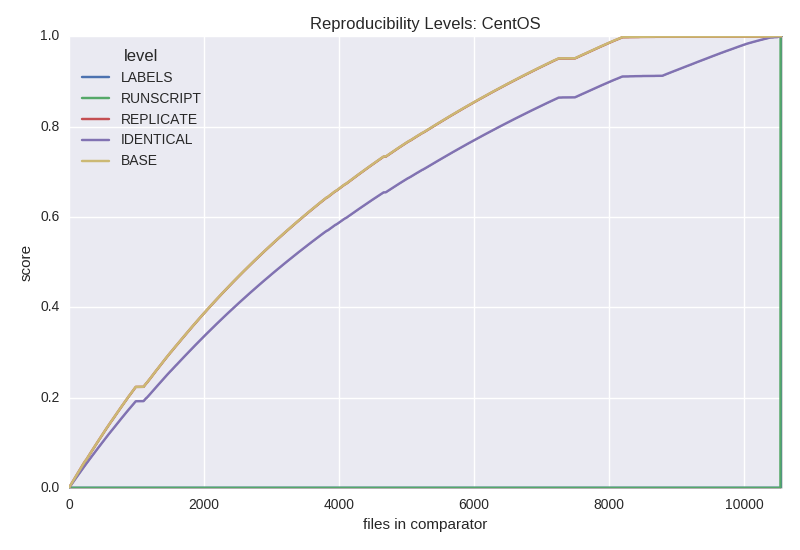

Supplement: S4 Fig — We calculated a comparison of the CentOS base operating system against all reduced versions of itself. (PNG) [file pone.0188511.s004.png]
